# Accuracy in diagnosing caries in young permanent molars using interproximal radiographic imaging and validation by artificial intelligence

**DOI:** 10.4317/jced.62396

**Published:** 2025-06-01

**Authors:** Débora Heloísa Silva de Brito, Thaysa Gomes Ferreira Tenório dos Santos, Samylla Glória de Araújo Costa, Adriana Stone dos Santos, Igor Lucas Balbino da Silva, Nathália Regina Cauás da Silva, Bruno José Torres Fernandes, Richard Niederman, Cláudia Cristina Brayner de Oliveira Mota, Márcia Maria Fonseca da Silveira, Mônica Vilela Heimer, Aronita Rosenblatt

**Affiliations:** 1 DDS, MSc, PhD student. University of Pernambuco, School of Dentistry – Department of Pediatric Dentistry, Recife, Pernambuco, Brazil; 2 DDS, MSc student. University of Pernambuco, School of Dentistry – Department of Pediatric Dentistry, Recife, Pernambuco, Brazil; 3 Undergraduate student. University of Pernambuco, Polytechnic School of Pernambuco – Department of Computer Engineering. Recife, Pernambuco, Brazil; 4 MSc student. University of Pernambuco, Polytechnic School of Pernambuco – Department of Computer Engineering. Recife, Pernambuco, Brazil; 5 DDS, PhD, Professor. University of Pernambuco, Polytechnic School of Pernambuco – Department of Computer Engineering. Recife, Pernambuco, Brazil; 6 DDS, PhD, Professor. Department of Epidemiology & Health Promotion, School of Dentistry, University of New York, New York, EUA; 7 DDS. PhD Adjunct Professor, School of Dentistry University of Pernambuco - Arcoverde, Pernambuco, Brazil; 8 DDS, PhD, Professor. University of Pernambuco, School of Dentistry – Department of Radiology., Recife, Pernambuco, Brazil; 9 DDS, PhD, Professor. University of Pernambuco, School of Dentistry – Department of Pediatric Dentistry, Recife, Pernambuco, Brazil

## Abstract

**Background:**

Caries lesions, in their early stages, can be challenging to identify clinically, as they often do not cause symptoms or are in areas that are difficult to access. Caries diagnosis involves high subjectivity and can lack consistency among professionals with different backgrounds and levels of experience. Discrepancies may occur between examiners or even with the same examiner at other times. With technological advancements, increasingly efficient methods for diagnosing dental caries are available, and new techniques and tools are under study. This study aims to evaluate the accuracy of diagnosing caries lesions in young permanent molars using interproximal radiographs by training object detection algorithms with an artificial intelligence (AI) system and comparing them to inter-examiner diagnoses.

**Material and Methods:**

A descriptive study was conducted in interproximal images of the first permanent molars of children aged between 6 and 9 years. The radiographs were obtained from three private radiological clinics in The Federal District The training was conducted by graduate dentists and calibrated using Professor of Radiology (MCF) as the gold standard. The YOLOv8 model architecture and a pre-trained classifier (EfficientNet-B0) were used.

**Results:**

The kappa agreement index was obtained to evaluate the degree of agreement between examiners. The inter-examiner agreement in the caries diagnosis was considered excellent, being 97.4%, with a kappa value of 0.88. The YOLOv8 model was applied to detect carious teeth using AI. The results show that the model achieved excellent performance, with accuracy metrics of 91% and precision of 98%. The EfficientNet-B0 classifier categorized teeth with and without caries lesions. The classifier achieved an accuracy of 89%.

**Conclusions:**

There was excellent inter-examiner agreement in evaluating caries diagnosis for the teeth assessed. The AI-based method proposed in this study showed good performance and proved effective in recognizing caries lesions in radiographic images.

** Key words:**Dental caries, Artificial intelligence, Radiography, Bitewing, Child.

## Introduction

The reduction of dental caries among children in industrialized countries in recent decades is a reality. However, it remains one of the most prevalent diseases in humans and affects millions of children worldwide (1). Although visual and tactile exams are the most accurate methods for diagnosing cavities, dental radiographs are essential auxiliary diagnostic tools (2). However, beyond the subjective clinical examination, examiners may fail to detect existing lesions, mainly located on proximal surfaces or in regions with complex anatomy, such as pits and fissures (3,4).

Permanent mandibular and maxillary first molars are the first teeth to erupt in the posterior part of the arch; they are often the most affected by caries due to their morphological characteristics, pits, and fissures (5-7). However, it is necessary to consider the limitations of the radiographic method regarding the non-diagnosis of caries lesions, especially lesions on proximal surfaces, due to the location of initial lesions in the enamel and minimal loss of hard tooth tissues (8,9).

The most recommended radiographic technique for detecting caries is the interproximal or bitewing, which provides information to complement the diagnosis, as it allows for a better estimation of the depth of proximal and occlusal caries lesions in dentin than clinical inspection alone (10). Due to the difficulty in diagnosing carious lesions, the World Health Organization (WHO) proposed the standardization of diagnostic criteria for evaluating oral health conditions through repeated exams on the same individuals by the same evaluators at different times, aiming to ensure the consistency of data obtained in different places and uniformity in the interpretation of exams, minimizing diagnostic discrepancies (11-13).

With the advancement of technology, increasingly efficient methods to diagnose dental caries have been studied, and new techniques and tools are being investigated and implemented in the market (14). Object detection is an artificial intelligence (AI) machine learning technique that identifies objects’ presence and location in images (15). The field of computer vision deals with modeling and replicating human vision through a machine. The central objective of computer vision is to interpret a 3D scene from 2D images, extracting the information in the structures present in the scene (16). AI has shown great potential to assist in diagnosing diseases and treatment planning in Dentistry (17-20). Machine learning for caries detection in dental radiographs is a promising method. Applying the convolutional neural network can establish an effective system for recognizing these diseases (21).

YOLO is a one-stage, high-precision object detection convolutional neural network developed by Ultralytics. It is based on the popular YOLO (You Only Look Once), created in 2016 (22). YOLOv8 is the eighth and most recent version, faster and more accurate than previous versions (23). Deep learning in X-ray images with this object detection algorithm can help dentists quickly and accurately identify dental caries lesions (21).

Given the above, this study aims to compare the accuracy of caries diagnosis in young permanent molar images through interproximal radiographs to the evaluation by AI machine learning based on the object detection algorithm YOLOv8 and a pre-trained EfficientNet-B0.

## Material and Methods

This is a cross-sectional descriptive study of secondary data of Interproximal radiographs from a database of three private radiology clinics in Brasília-DF. The teeth right permanent maxillary first molar, left permanent maxillary first molar, left permanent mandibular first molar and right permanent mandibular first molar of children aged six and one months to 9 years and 11 months were examined. The database of the three dental clinics, specialized in dental radiology, stored approximately 5,000 images of the first permanent molars; however, only 500 met the inclusion criteria: interproximal radiographs of the right and left sides, showing the presence of the four first permanent molars, of children aged 6 to 9 years of both sexes.

The evaluated areas on the radiographs were the mesial and distal proximal surfaces and the occlusal surface. The radiographs were selected according to inclusion and exclusion criteria.

The inclusion criteria were radiographs of children aged 6 to 9 years of both sexes, complete radiographic records of the patient, including interproximal radiographs of the right and left sides, and images of the first four permanent molars. The exclusion criteria were the presence of dental anomalies in shape, number, and structure; teeth with incomplete images; endodontically treated teeth; radiographs showing trabecular bone outside standard patterns; and radiographs with distortions or imperfections that hinder radiographic interpretation.

**Figure 1:**
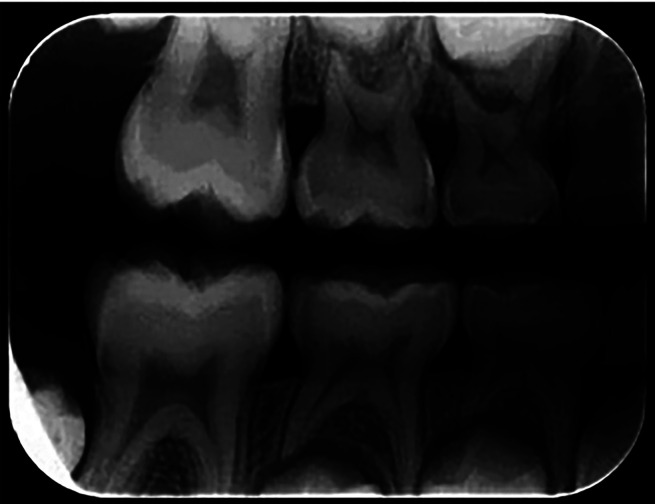
Right.

**Figure 2:**
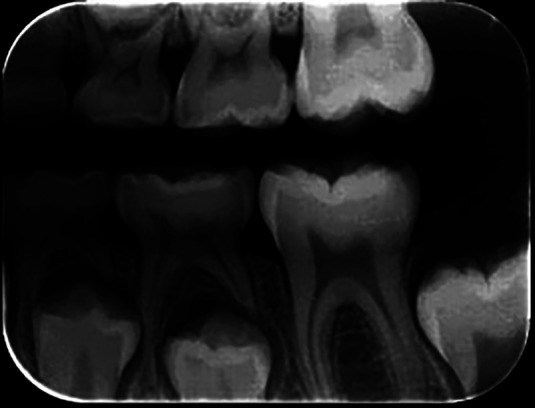
Left.

The training was conducted with the examiners, using Professor MCF, a radiology and diagnosis expert with over 20 years of experience issuing radiographic reports as a gold standard. The examiners received written and verbal instructions on how to analyze the radiographs. One radiograph was evaluated at a time, with a maximum of 20 images per day, to avoid compromising the assessments due to visual fatigue, with a minimum interval of five days. The radiographic images were evaluated in a predetermined, dimly lit environment. The interproximal radiographs were analyzed for the presence or absence of caries. Figures 1 and 2 show examples of analyzed radiographs (right and left side).

After the training, the examiners analyzed 60 radiographs (pilot study). The Kappa test was used to assess inter-examiner agreement (calculation based on agreement between the examiners), considering the following values: <0 (no agreement); 0-0.20 (minimal agreement); 0.21-0.40 (fair agreement); 0.41-0.60 (moderate agreement); 0.61-0.80 (substantial agreement); 0.81-1.0 (perfect agreement) (24). The examiners were considered calibrated when the Kappa test values were between 0.61 and 1.0 (substantial to perfect agreement). The agreement between the examiners observed in the pilot study was 91.7%, with a Kappa value of 0.68 (good agreement).

In interproximal radiographic images, dental caries can be recognized by the radiolucency of the enamel and dentin in the tooth structure. Single-tooth radiographs were cropped from interproximal radiographic images using the YOLOv8 object detection model to locate each tooth accurately. The YOLOv8 architecture consists of convolutional layers and pooling operations, which transform the input image into a series of feature maps of different sizes. These maps are then used to detect objects at various scales and resolutions, utilizing bounding box detection techniques, defined as rectangles that enclose objects of interest in the image, and object classification. YOLOv8 can be described as a combination of three components: the backbone network, the neck network, and the detection network.

Of the 500 radiographs analyzed, 152 contained teeth with carious lesions. These 152 images of young molar teeth were used for training the tooth identifier with the YOLO network. The images were manually annotated using the Labeling tool, creating bounding boxes around each visible tooth. The annotated images underwent several data augmentation processes to expand the existing information base, increase the variability of the dataset, and improve the model’s robustness.

The YOLO network, specifically the YOLOv8 version, was chosen for tooth detection due to its efficiency in real-time detection tasks. The training was conducted using the 152 annotated images over 2,000 epochs. The auto batch size was used to optimize the data load during training. Various hyperparameters, such as the learning rate, were adjusted interactively to maximize the model’s performance.

After achieving good accuracy in tooth detection, the trained YOLOv8 model was used to infer 152 labeled radiographs with carious lesions. The radiographs were analyzed and labeled by two examiners, and the radiographs labeled by the most experienced dentist were selected. Specific code was developed to perform image inference using YOLO, crop the regions of interest (teeth), and separate these images into distinct folders: one containing teeth with carious lesions and another containing teeth without lesions. The separation was based on analyzing the intersections between the bounding boxes generated by YOLO and the caries bounding boxes provided by the dentist, ensuring the correct categorization of the teeth.

The images of the teeth, already separated into categories with and without carious lesions, were used to train a carious lesion classifier. The EfficientNet-B0, a convolutional neural network architecture known for its efficient performance and high accuracy in image classification tasks, was chosen. The EfficientNet-B0 classifier was trained using the dataset, with the teeth adequately categorized. After training, the model achieved good accuracy, demonstrating robust performance in classifying carious teeth.

The research ethics committee approved the project. The observed agreement value, the kappa agreement index, and respective confidence intervals for the population kappa were obtained to assess the degree of agreement between the evaluators. To evaluate differences between faces, sexes, and age groups, the Generalized Estimation Equations (GEE) procedure was used, considering repetitions of face and tooth per subject. This procedure allows modeling the data structure with more than one observation per scanned image (more than one face and more than one tooth per patient) and helps reduce the bias of possible correlation between the results of the same subject. The level of significance used was 5.0%. The data were entered into an Excel spreadsheet, and the program used for statistical calculations was IMB SPSS version 25.

## Results

The age of the 250 children whose right and left radiographs were analyzed ranged from 6.08 to 9.92 years, with a mean of 8.09 years, a standard deviation of 1.04 years, and a median of 8.08 years. The lowest percentage corresponded to those aged 6 to 6.99 (15.6%), and the percentages of the other three age groups ranged from 23.6% to 30.8%. Most participants (56.0%) were female ( Table 1).

The observed agreement percentages between the evaluators for the total sample studied (500 radiographs, 1,000 teeth, and 3,000 surfaces) ranged from 96.5% to 98.0%, and the kappa values ranged from 0.85 to 0.89 (Excellent agreement). Overall, the observed agreement was 97.4%, and the kappa was 0.88 (Excellent agreement) ( Table 2).

The prevalence of caries on each tooth and overall was higher on the occlusal surface than on the mesial and distal surfaces, especially on teeth 36 and 46. The incidence of caries on tooth 36 was 25.7% on the occlusal surface and 0.4% on each of the other two surfaces; on tooth 46, it was 23.3% on the occlusal surface and ranged from 0.4% to 0.8% on the other two surfaces. On tooth 16, the prevalence of caries was 6.4% on the occlusal surface and ranged from 1.0% to 1.2% on the other two surfaces, and on tooth 26, the percentage was 9.2% on the occlusal surface and ranged from zero to 1.6% on the other two surfaces. For all four teeth, the rate of caries was 16.5% on the occlusal surface and ranged from 0.5% to 1.0% on the other two surfaces. Significant differences were recorded between the surfaces for each of the teeth and in the total group of teeth ( Table 3).

**Figure 3:**
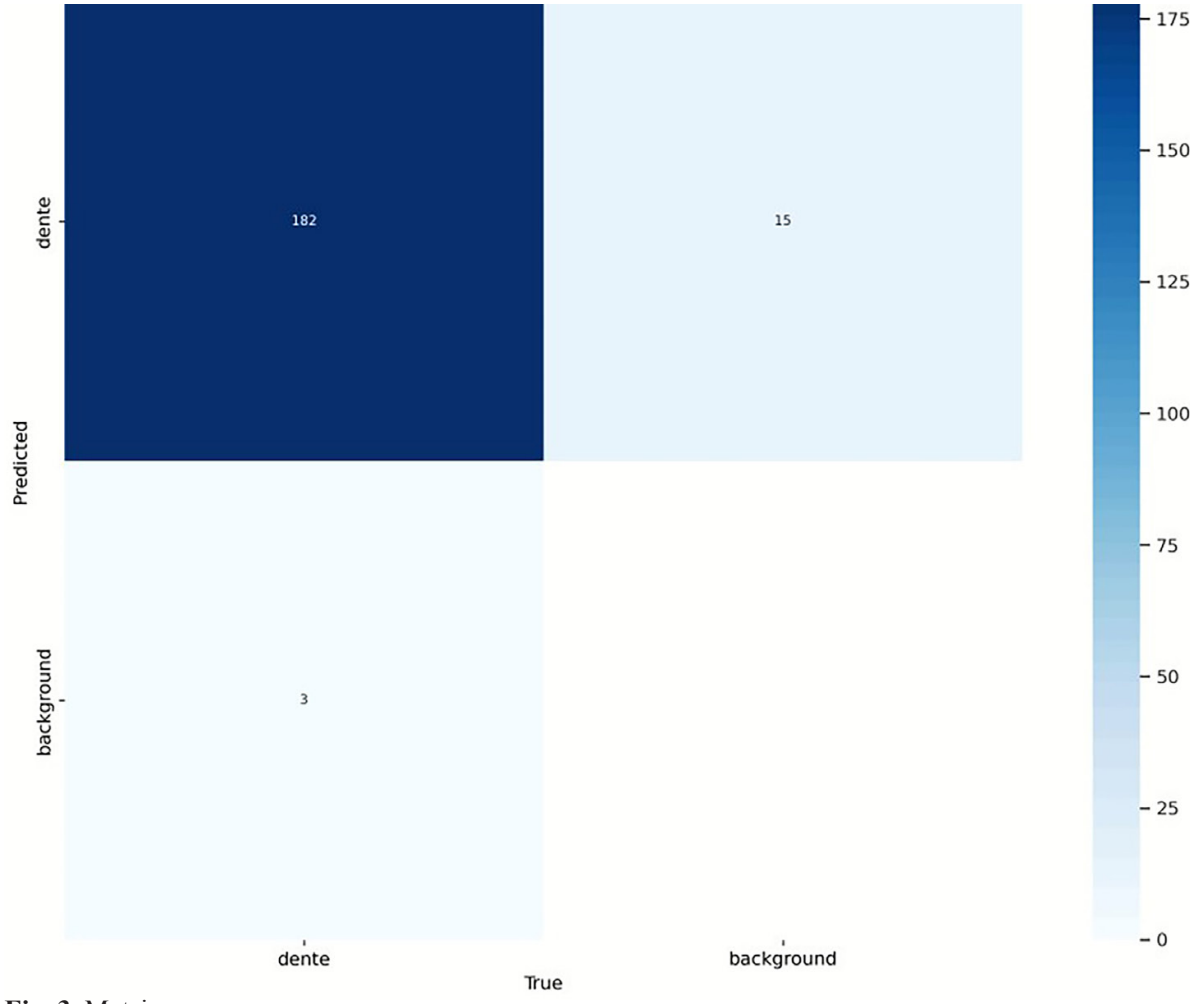
Matrix.

Regarding the detection of teeth using AI, the model validation was performed using the YOLOv8 network with a set of 31 images (20% of the sample), selected from 152 images of young permanent molar teeth, all containing initial-stage caries lesions. The validation was carried out using a confusion matrix, which analyzed true positives (tooth/tooth): 182, false negatives (tooth/background): 15, false positives (background/tooth): 3, and true negatives (background/background): not applicable (no background labels in the matrix). The confusion matrix presented in Figure 3 summarizes the model’s performance.

The results demonstrated that the YOLOv8 model achieved excellent performance in detecting carious teeth, with very few false positives and false negatives. Based on the confusion matrix, the following performance metrics were calculated: Accuracy: 91% and Precision: 98%. These metrics confirm the high precision of the model in tooth detection.

After detecting the teeth, the regions of interest were classified as teeth with caries lesions and teeth without caries lesions using the EfficientNet-B0 classifier. The database was divided into 80% for training and 20% for validation, using a stratified split strategy to maintain the class proportions in both splits.

The classifier’s accuracy was 89%, highlighting the model’s effectiveness in distinguishing between teeth with and without caries lesions despite the quality of the images and the fact that the lesions were in the initial stage. The methodology adopted in this research, using YOLOv8 for tooth detection and EfficientNet-B0 for caries lesion classification, proved to be a practical approach for evaluating dental images.

## Discussion

The kappa index can be considered a measure of agreement for calculating intra/inter-examiner reproducibility, which is highly important during the calibration of dental caries diagnosis, as it generates quality information and is an adjusted agreement index that considers the proportion attributed to chance (25). Dias *et al*. (2010), in their study on the accuracy of direct digital radiography in detecting occlusal caries in deciduous teeth compared to conventional radiography and visual inspection, observed that inter-examiner reproducibility in caries detection ranged from substantial to almost perfect with traditional radiography (0.770 to 0.919), digital radiography (0.686 to 0.883), and visual inspection (0.666 to 0.878) (26).

In the study by Signori (2018), three experts in cariology and restorative dentistry from different universities were invited to analyze a series of interproximal radiographs. The inter-examiner agreement between them, based on the Kappa index, was substantial (kappa > 0.60) to excellent (kappa > 0.86) concerning the diagnosis. In this study, Kappa revealed significant inter-examiner agreement across all teeth and surfaces analyzed, considered an excellent concordance (27). This indicates the importance of examiner calibration in enabling them to participate in data collection for caries diagnosis in epidemiological surveys (12).

In the study by Caceda *et al*. (2023), involving 160 children aged 7 to 12 years, evaluating the occlusal surface of 632 first permanent molars, the mean and standard deviation of sensitivity using ICDAS II were observed to be 0.47 ± 0.16, respectively. When specificity was calculated for the first permanent molars, it was 0.81 ± 0.13 respectively. The study considered these high values for detecting caries in the enamel or dentin of the first permanent molars (28). In this research, the prevalence of caries in each tooth and overall was higher on the occlusal surface than on the mesial and distal surfaces, especially in teeth 36 and 46, corroborating the study by Botelho *et al*. (2011), where the lower first permanent molars were the most affected by caries, with no significant difference found between the right and left sides in the occurrence of carious lesions (7).

The study by Lee *et al*. (2018) focused on detecting dental caries in a single tooth through cropped images based on deep learning for premolars and molars in periapical radiographs. The diagnostic accuracies for the caries prediction model for premolars and molars were 89.0% and 88.0%, respectively (29). This study’s method used to diagnose dental caries achieved similar accuracy, with the pre-trained EfficientNet-B0 model providing 89% accuracy.

In a study on deep learning-based dental caries recognition from dental X-ray images, where models and metrics were evaluated, EfficientNet-B0 demonstrated the highest average accuracy, reaching 94.94% for dental caries. According to the author, this metric indicates that EfficientNet-B0 outperforms other models assessed in their study and exhibits robust performance and strong predictive capabilities in accurately classifying carious lesions (21). EfficientNet-B0 was chosen as the CNN model in the present work and performed well in identifying dental caries.

In a systematic review conducted by Ragab *et al*. (2024), using the PubMed database to identify articles reviewed between 2018 and 2023, relevant studies were found employing YOLO for various tasks, including detecting carious lesions. These findings demonstrated the efficacy of YOLO in surpassing other existing methods for these tasks (30). Based on the results presented in the current study, it was also found that it is possible to train an object detection model for the given task (caries detection in radiographs). However, it was observed that the relative success of the algorithm might also depend on the pre-processing methods applied to the data, such as the YOLOv8 model, which applies various pre-determined transformations to the images, generating new representations of the input features.

The following methodology, which combined tooth detection with YOLOv8 and carious lesion classification with EfficientNet-B0, proved effective in analyzing dental radiographs with early-stage carious lesions. The technique overcame limitations such as image quality and more direct approaches in training models for detecting carious lesions in radiographs, resulting in a robust and accurate system for identifying and classifying teeth with these lesions.

## Conclusions

1. Excellent inter-examiner agreement was achieved in assessing caries diagnosis for all evaluated teeth.

2. The method based on artificial intelligence technology using YOLOv8 and EfficientNet-B0 achieved high performance and proved effective for recognizing dental caries lesions in radiographic images.

3. Good accuracy in teeth with and without carious lesions is essential for the methodology’s feasibility. This will significantly contribute to the advancement of automated techniques in dental diagnosis, aiming to minimize and automate the subjective diagnostic process performed by humans.

## Figures and Tables

**Table 1:** Evaluation of the Demographic Profile.

Variable	n (%)
TOTAL	250 (100,0)
Age Range (years)	
6 to 6,99	39 (15,6)
7 to 7,99	77 (30,8)
8 to 8,99	75 (30,0)
9 to 9,99	59 (23,6)
Sex	
Male	110 (44,0)
Female	140 (56,0)

**Table 2:** Assessment of Inter-Rater Agreement.

Variable	Sample	Observed Agreement		Kappa	95% Confidence
		n	%		Interval
Tooth 16	750	731	97,5	0,89	0,84 a 0,94
Tooth 46	750	735	98,0	0,89	0,84 a 0,95
Tooth 26	750	724	96,5	0,85	0,80 a 0,91
Tooth 36	750	731	97,5	0,87	0,82 a 0,93
General	3000	2921	97,4	0,88	0,85 a 0,90

**Table 3:** Assessment of caries prevalence by surface per tooth and overall for the 4 teeth.

	Surface				
Variable	Mesial	Occlusal	Distal	Total group	p-value
	n (%)	n (%)	n (%)	n (%)	
Tooth 16					p^(1)^ = 0,003*
Presence of caries	3 (1,2)	15 (6,4)	2 (1,0)	20 (3,0)	
Absence of caries	240 (98,8)	218 (93,6)	197 (99,0)	655 (97,0)	
TOTAL	243 (100,0)	233 (100,0)	199 (100,0)	675 (100,0)	
Tooth 26					p^(1)^ < 0,001*
Presence of caries	4 (1,6)	21 (9,2)	-	25 (3,7)	
Absence of caries	242 (98,4)	207 (90,8)	205 (100,0)	654 (96,3)	
TOTAL	246 (100,0)	228 (100,0)	205 (100,0)	679 (100,0)	
Tooth 36					p^(1)^ < 0,001*
Presence of caries	1 (0,4)	64 (25,7)	1 (0,4)	66 (9,0)	
Absence of caries	249 (99,6)	185 (74,3)	236 (99,6)	670 (91,0)	
TOTAL	250 (100,0)	249 (100,0)	237 (100,0)	736 (100,0)	
Tooth 46					p^(1)^ < 0,001*
Presence of caries	2 (0,8)	58 (23,3)	1 (0,4)	61 (8,3)	
Absence of caries	248 (99,2)	191 (76,7)	233 (99,6)	672 (91,7)	
TOTAL	250 (100,0)	249 (100,0)	234 (100,0)	733 (100,0)	
Total 4 dentes					p^(2)^ < 0,001**
Presença de cárie	10 (1,0)	158 (16,5)	4 (0,5)	172 (6,1)	
Absence of caries	979 (99,0)	801 (83,5)	871 (99,5)	2651 (93,9)	
TOTAL	989 (100,0)	959 (100,0)	875 (100,0)	2823 (100,0)	

(*) Significant Difference at the 5.0% Level (**) By Chi-Square, using the repeated measures model per tooth through the GEE function in the comparison between surfaces.

## Data Availability

The datasets used and/or analyzed during the current study are available from the corresponding author.
